# Medical Exhibitions

**Published:** 1908-11-14

**Authors:** 


### Medical Exhibitions.

In modern times it is increasingly recognised that the chief aim of medicine is not so much curative as prophylactic. We are coming back, in fact, to the theories of the Imhotepian priests who wrote upon their doorways the mystic word "guard"—a motto in which we prefer to see not the narrow prejudice of sect that punished with death the violation of professional secrecy, but the careful regard of a parental priesthood for the welfare of a nation. Such an interpretation is not inconsistent with what we know of the history of the Egyptian medical priests. They invested their science, it is true, with all the mystery of an Eleusinian procession, but they also popularised thereby the main essentials of hygiene and public health. The art of curing disease was the major mystery; the greater art of prophylaxis they gave away freely, with a mouthful of prayer and a scroll of incantation, to every member of the multitude. The Imhotepian temple became the first domicile of an institution, which has continued in some shape or form ever since. There were displayed "models of good dwelling-houses, pure food and fine drink," which, as Ebers remarks, are matters that the people could see and hear about without a shudder of disgust or a thrill of fearful anticipation. The exhibition of instruments was carefully avoided, the secrets of the craft were never revealed; for the object of these temple displays was to educate the people to guard their own welfare, not to dabble in the grave problems of pathology and therapeutics.

The originators of our periodical modern medical exhibitions would do well not to lose sight of the Egyptian example. It is only by object lessons that the public learns to remember. We may talk and write volumes on national deterioration and racial efficiency, encyclopædias on the care of health, and whole libraries on the abuse of this or that fad: such labours will only be appreciated by a small minority, just as in the infant school it is the few who can grasp theory and the spoken word, unassisted by the aids of form and colour. The majority needs a kindergarten system throughout life; and when we meet with a section that demands more, it raises the suspicion which we feel when the precocious child comes forward—that over-early development too often spells premature degeneration. At the same time there are certain things in which the public may legitimately claim instruction. The development of our knowledge of the cancer problem, for example, and the advances of our methods of prevention against the spread of infectious diseases, while they are directly of professional interest alone, are indirectly the concern of the layman as much as of his medical attendant, and a sane and lucid exposition of them is desirable.

To admit the reasonableness of such claims and to attempt to supply the wants of the public should be the aim of the promoters of medical exhibitions that are not strictly confined to the profession. As an educative factor, we are inclined to put the popular medical exhibition which is really instructive and not a mere collection of pills and powders, mineral waters and malt extracts, on a very high level. Its educative possibilities are immense, and as there is reason to believe that it is a popular institution, it may do considerable good by drawing public attention to certain much needed measures of reform. At present there is about such exhibitions just that element of mystery and morbidness that makes them doubly interesting to the man in the street, for whom all things medical have a grim fascination. The individual who would not trouble himself to attend a hygienic exhibition will cheerfully travel miles to look at a medical collection. The attraction, we fear, is the morbid element which is popularly associated with such displays; but no apology is needed for taking advantage of that interest and using it to further the advance of useful knowledge amongst the community.

